# The impact of physical activity on inhibitory control of adult ADHD: a systematic review and meta-analysis

**DOI:** 10.7189/jogh.15.04025

**Published:** 2025-03-14

**Authors:** Yi Yang, Chang-Hong Wu, Liang Sun, Ting-Ran Zhang, Jiong Luo

**Affiliations:** 1 Southwest University Sports Institute Sports Rehabilitation Centre, Chongqing, China; 2 Wuhan Sports University School of Sports Medicine, Wuhan, China

## Abstract

**Objective:**

We aimed to Investigate physical activity's effects on inhibitory control in adult attention deficit hyperactivity disorder (ADHD).The benefits of physical activity on the inhibitory control of adult ADHD were explored in the hope of providing some suggestions for approaches to treating adult ADHD.

**Methods:**

We searched the databases PubMed, Web of Science, CNKI and Wanfang for randomised controlled trials (RCTs) of the effect of physical activity on inhibitory control in adults with ADHD, using PRISMA guidelines. We used used the Cochrane Bias Risk Assessment Criteria to assess the methodological quality of the included studies. Finally, we performed a heterogeneity analysis and a potential publication bias analysis using Revman 5.4.

**Results:**

A total of eight articles, 14 studies, and 373 experimental subjects were included in the systematic review and meta-analysis. The meta-analysis results showed that both acute exercise (standard mean deviation (SMD) = −0.65, 95% confidence interval (CI) = −1.10,−0.2, *P* = 0.005) and chronic exercise (SMD = −1.77, 95% CI = −2.84, −0.69, *P* = 0.0001) have a positive effect on the inhibitory control of adult ADHD. Pilates (SMD = −2.22, 95% CI = −2.97, −1.47, *P* < 0.0001), Tai Chi (SMD = −2.20, 95% CI = −6.25, −1.8, *P* = 0.25), cycling (SMD = −0.67, 95% CI = −1.27, −0.08, *P* = 0.03), vibration training (SMD = −0.67, 95% CI = −1.39, −0.05, *P* = 0.07), yoga (SMD = 0.01, 95% CI = −0.50, −0.48, *P* = 0.97), and other different exercise styles have significant differences in their effects on adult ADHD inhibitory control.

**Conclusions:**

Physical activity has a beneficial effect on inhibitory control in adults with ADHD. However, more research is needed to examine the beneficial effects of different types of physical activity, intervention modalities, and dose-response effects of intensity.

**Registration:**

This review was registered with INPLASY (registration number: 202490109).

Attention deficit hyperactivity disorder (ADHD) is often considered a neurodevelopmental disorder characterised by symptoms of inattention, hyperactivity, and impulsivity [1]. According to statistics, the global prevalence of persistent adult ADHD in 2020 was 2.58%, and the prevalence of symptomatic adult ADHD was 6.76% [2]. Attention deficit hyperactivity disorder is a complex disease, and according to a global study, 23% of ADHD patients also suffer from comorbidities. It mainly includes emotional disorders, anxiety disorders, substance use disorders, and other behavioural disorders (such as impulse control disorders, and antisocial personality disorders) [3,4]. As is well known, ADHD is one of the most common neurodevelopmental disorders in early childhood [5]. However, the effects of ADHD extend beyond childhood and into adulthood, with approximately one-fifth of adult patients still meeting the diagnostic criteria for the disease. Patients still suffer from the three core problems of difficulty concentrating, hyperactivity, and impulsive behaviour, which are often closely related to various clinical mental health problems and social dysfunction, posing major challenges in their lives. [6,7].

In addition, ADHD patients often suffer from cognitive impairment. For example, executive dysfunction may lead to poor inhibitory control in adult ADHD, making it prone to impulsive or hyperactive behaviour [8]. Executive function is one of the core functions of cognitive function. The executive function includes inhibitory control, cognitive flexibility, and working memory. Inhibitory control refers to a person's ability to overcome automatic or strong responses to stimuli. Inhibition ability deficiency can lead to a decrease in an individual's ability to respond to external stimuli, which may impair functional independence and safety, and is known as a hallmark of executive dysfunction in ADHD patients [9]. In addition, adult ADHD also has various impairments in attention and memory, and its performance in spatial working memory, planning, and transfer focus tests is significantly worse than that of generally healthy adults [10]. Patients may have difficulty resolving obstacles such as self-regulation, self-organisation, and difficult processing due to deficits in spatial memory [11]. Long term adjuvant therapy is more helpful in improving adult ADHD symptoms and cognitive deficits. At present, research on adult ADHD mainly uses drug therapy [12], cognitive behaviour therapy [13], mindfulness training [14]. These different intervention methods have shown improvement effects on the symptoms of adult ADHD. However, the benefits of improving cognitive function, and social and behavioural problems in ADHD patients are not significant [3,15]. The positive effects of exercise on brain cognition and social behaviour have been widely confirmed. Therefore, exercise therapy can serve as a potential treatment for improving cognitive function in adult ADHD.

Numerous studies have shown that physical activity has a positive effect on ADHD symptoms [16,17]. Meanwhile, exercise intervention also has improvement effects on cognitive function, emotional state, mental health, and physical fitness indicators of ADHD patients [18]. Unfortunately, most studies have focused on children and adolescents, and there is relatively little research on how physical activity improves adult ADHD. In addition, physical activity can enhance cognitive function and may be a protective factor for ADHD patients, with a significant improvement in inhibitory control [19]. After participating in 30 minutes of acute aerobic exercise, 20 children with ADHD found that vigorous exercise promoted their Stroop test performance. The preliminary explanation for the exercise effect is that exercise allocates attention resources, affects the dorsolateral prefrontal cortex, and is associated with exercise-induced dopamine release [20]. A single aerobic intervention can help shorten the reaction time and increase P3 amplitude in children with ADHD and also have a positive impact on cognitive functions such as inhibitory control and working memory [21]. In addition, 12 consecutive weeks of swimming intervention can also help improve selective attention and inhibitory control levels in children with ADHD [22]. From this, it can be seen that physical activity is a potential treatment for the prevention and treatment of attention deficit hyperactivity disorder patients. Especially for the improvement of inhibitory control, attention performance, impulsivity, and hyperactivity in patients. However, some review studies suggest that although single physical activity has a strong impact on attention and inhibitory control, the results on the further symptoms of ADHD in adults are mixed. Meanwhile, there are few randomised controlled trials on long-term physical activity and they have shown high heterogeneity. Additionally, acute aerobic exercise improves impulsive behaviour in adults with ADHD, but cannot improve other cognitive-related manifestations [23]. Based on this, this article will use meta-analysis to organise the latest literature on the relationship between physical activity and cognitive function in adult ADHD. Exploring the benefits of physical activity in inhibiting and controlling adult ADHD, and providing some suggestions for the treatment of adult ADHD.

## METHODS

This study strictly followed the requirements of PRISMA 2020 for literature screening, inclusion, data processing, and analysis [24]. The study has been registered with INPLASY (registration number: 202490109).

### Search strategy

Two researchers utilised databases such as PubMed, Web of Science, Embase, Cochrane Library, China National Knowledge Infrastructure (CNKI), and Wan Fang. According to the Population, Intervention, Comparison, Outcomes and Study (PICOS) principle, the search terms include adult ADHD, ADHD symptoms, physical activity, exercise intervention, exercise therapy, cognitive function, executive function, inhibitory control, and RCT. Simultaneously conducting a search using ‘subject words + free words’.The literature search period is from 1 January 2000 to 1 September 2024.

#### Inclusion and exclusion criteria

The PICOS principles and model were used to design the screening, inclusion and exclusion criteria for the study.

Inclusion criteria:

1) The research subjects consist of adults aged 18 and above.

2) The experimental group has a strict exercise prescription design, and the intervention methods include various types of physical activities, including game activities, sports activities, physical activities, and fitness activities. The control group can participate in daily activities or not intervene.

3) The design of exercise prescriptions follows the standards of the American College of Sports Medicine (ACSM).

4) The experimental research design should be a randomised controlled trial (RCT) study.

5)The outcome measure is inhibitory control.

#### Exclusion criteria:

1) Non-English or non-Chinese literature.

2) Non-experimental studies and control group studies without pre- and post-tests.

3) Literature unrelated to exercise prescription, adult ADHD, and cognitive function.

4)Review, thesis, conference paper, qualitative research, and literature for which research data are not available. To ensure the quality of the study, reduce bias in the conclusions, and increase the comparability of the samples, we chose journal articles rather than conference papers and dissertations because journal articles are more rigorously reviewed and have more in-depth and comprehensive content, whereas the rapid publication of conference papers can lead to unstable conclusions and large differences between different types of papers.

### Literature screening and evaluation

**Figure 1:**
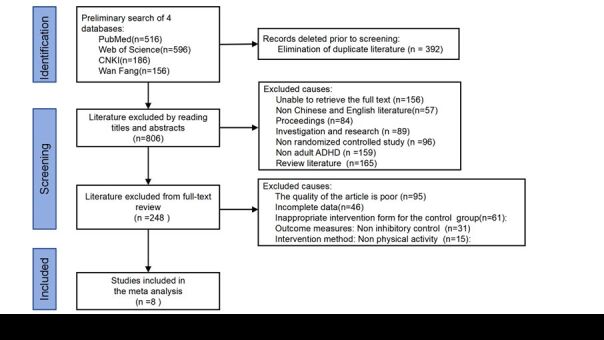
Literature retrieval and screening process.

Use NoteExpress software to reduce the weight of the retrieved studies. Two researchers independently conducted studies screening by reading the titles and abstracts of the studies for preliminary screening and then reading the full texts of the studies that may be included. After the studies selection is completed, a third party will compare and organise the studies database. Based on the search results, 342 Chinese studies and 1212 English studies were retrieved, totaling 1454 studies. Remove 392 duplicate articles from the studies management software. By reading the title and abstract selection by two authors, they need to reach a consensus. If there are still differences after discussion, seek the opinion of the third reviewer. By reading the title and abstract, 806 references were removed, and by reading the full text, 248 references were removed. Finally, eight articles were included in the studies (**Figure 1**).

### Study characteristics

The included studies were encoded using Excel, and extracted feature values including author, publication year, nationality information, sample characteristics (age and sample size), and physical activity intervention dose (intervention method, intervention period, single exercise duration, and frequency) (**Table 1**).

**Table 1:** Characteristics of the included studies

Study details	Country	Age	Total sample size (N)	Sample size (n)		Intervention cycle	Single duration	Physical activity mode\intensity	Intervention frequency	Outcome indicators
				**Experimental group (female)**	**Control group (female)**					
Mehren et al. [25], 2019a	Spain	19–40	40	20(4)	20(5)	*	Exhaustion, no more than 30 min	Cycle ergometer, 50–70% HRmax	†	Flanker Task, visual Task
Mehren et al. [26], 2019b	Spain	21–41	20	23(3)	23(4)	*	Exhaustion, no more than 30 min	Cycle ergometer, 50–70% HRmax	†	Go/no-go task, fMRI test
LaCount et al. [27], 2022	America	19–22	36	18(9)	18(9)	*	16minute	Cycle ergometer, 85% HRmax	†	AX-CPT
Kuo et al. [28], 2024	China	20–27	52	26(10)	26(10)	*	30minute	Cycle ergometer, 50–70% HRmax	†	Stop signal task
Fuermaier et al. [29], 2014	the Netherlands	18–31	100	17(9)	83(43)	*	8minute	Vibration training	†	Stroop test
Fritz et al. [30], 2022	America	18–24	32	16(-)	16(-)	6week	60minute	Yoga	Twice a week	Flanker task,DCCS test
Converse et al. [31], 2020	America	18–23	21	9(-)	10(-)	7week	60minute	Tai Chi	Twice a week	Flanker task, DCCS test
Kouhbanani et al. [32], 2022	Iran	20–50	52	25	27	24week	45minute	Pilates	Three times a week	WCST, AX-CPT

AX-CPT – AX-Continuous Performance Test, DCCS – Dimensional Change Card Sort test, WCST – The Wisconsin Card Sorting Test

*One-time exercise.

**†**No relevant information has been provided.

The eight included studies were all randomised controlled trials conducted before and after testing. From the basic information of the research subjects, there were a total of 372 participants, with a minimum of five and a maximum of 25 participants. Two articles did not specify the gender characteristics of the subjects. The age range of the subjects is between 18 and 50 years old. From the perspective of the physical activity types and dosage parameters designed in the study, the 11 articles were divided into acute exercise and long-term exercise based on the duration of physical activity intervention. Among them, five articles are on acute exercise [25–29], three articles on long-term exercise [30–32]. The duration of a single intervention should not exceed 60 minutes. The intervention period for long-term exercise is between six and 24 weeks. Interventions in physical activity include cycling [25–28], vibration training [29], yoga [30], tai chi [31], pilates [32]. Most studies use measurement methods such as the Stroop Test, Flanker Task, and Go/No Go Task to examine the cognitive function of adult AHDH. The corresponding outcome measure is a continuous variable, therefore, the standard mean deviation (SMD) is selected as the effect measure.

### Quality assessment

**Figure 2:**
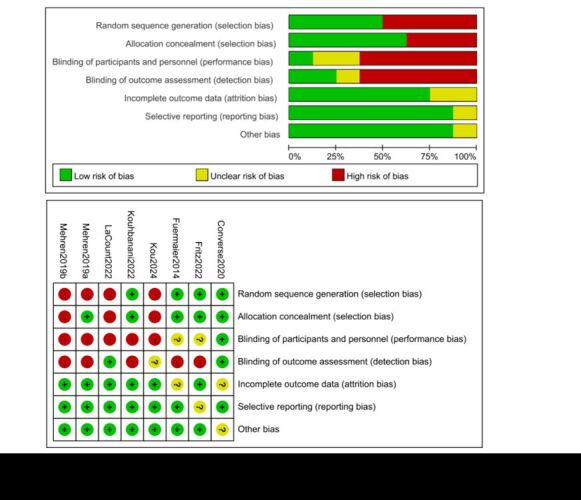
Risk ofbias assessment for randomised controlled trials

Using the Cochrane systematic review's ‘bias risk assessment’ tool to evaluate the quality of the included studies. The results of the Studies quality evaluation found that there was a certain degree of bias in the eight included articles, and the main reason for the bias was that the experiment failed to achieve double-blind. This is because it is difficult to blind the subjects and staff in exercise intervention (**Figure 2**).

### Statistical analysis

We used Review Manager 5.4 software to analyse the outcome measures of the eight included RCTS. The results of this study are all continuous variables, and different tools such as the Stroop Test and Flanker Task were used to test the outcome indicators. Therefore, the SMD was used to calculate the effect size, with a 95% confidence interval (CI) and *P* ≤ 0.05 indicating statistical significance.

## RESULTS

### Meta-analysis results

#### Meta-analysis of the impact of physical activity on inhibitory control of adult ADHD

**Figure 3:**
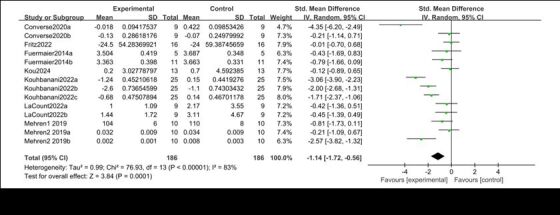
Meta analysis forest plot of the effect of physical activity on inhibitory control of adult attention-deficit/hyperactivity disorder.

Eight articles were included, and 14 studies reported the intervention benefits of physical activity on the inhibitory control of adult ADHD, involving a total of 372 participants. The results of the heterogeneity test showed *I*^2^ = 83%, so the random effects model was chosen (**Figure 3**). The meta-analysis results showed SMD = −1.14 (*P* = 0.0001), 95% CI = −1.72, −0.56. The results indicate that the combined effect of multiple studies has reached a high level and is statistically significant. The data shows that the inhibitory response time level of adult ADHD in the physical activity group is significantly higher than that in the non-exercise control group, revealing that physical activity can effectively improve the inhibitory control ability of adult ADHD.

### Subgroup analysis results

#### Subgroup analysis of the effects of different types of physical activity on ADHD inhibitory control

This study conducted a subgroup analysis on the inhibitory control of adult ADHD through two different types of physical activity: acute exercise and chronic exercise. Two variable groups included a total of 372 individuals, including 154 in the acute exercise group and 218 in the chronic exercise group. The sub-group analysis results showed a high degree of heterogeneity in the combined effect size between the two groups (*I*^2^ = 83%), indicating that different types of exercise have a certain impact on the relationship between physical activity and ADHD inhibitory control. The acute exercise group positively affected adult ADHD suppression control (SMD = −0.65, 95% CI = −1.10, −0.2, *P* = 0.005). This result is statistically significant. Second, the chronic exercise group had the greatest effect on the inhibition and control of ADHD (SMD = −1.77, 95% CI = −2.84, −0.69, *P* = 0.001). Meta-analysis is shown in Figure S1 in the **Online Supplementary Document**.

A total of 404 subjects were enrolled in different groups of physical activity modalities. We analysed cycling, vibration training, yoga, tai chi and pilates as subgroups with different variables. The sub-group analysis results showed that the combined effect sizes of each combination were highly heterogeneous (*I*^2^ = 84%), indicating that the intervention project had a certain impact on the control of physical activity and ADHD inhibition. Among them, the Pilates and Tai Chi groups showed the greatest effect in improving inhibitory control. The effect size of Pilates is SMD = −2.22, 95% CI = −2.97, −1.47, *P* < 0.0001,and the results show statistical significance. The effect size of the Tai Chi group is SMD = −2.20, 95% CI = −6.25, 1.8, *P* = 0.25, and the results are not significant. Second, there is not much difference in the effect size between the bicycle group and the vibration training group. The effect size of the bicycle group was SMD = −0.67, 95% CI = −1.27, −0.08, *P* = 0.03, and the results show statistical significance. The effect of vibration training is SMD = −0.67, 95% CI = −1.39, −0.05, *P* = 0.07, the results are not significant. The yoga group has the smallest effect size (SMD = 0.01, 95% CI = −0.50, −0.48, *P* = 0.97), the result is not significant. Meta-analysis is shown in Figure S2 in the **Online Supplementary Document**.

### Publication bias test

The overall results indicate that the funnel plot is relatively asymmetric, suggesting that there may be some publication bias in the results of this study (Figure S3 in the **Online Supplementary Document**).

### Sensitivity analysis

Sensitivity analysis was conducted on the eight included articles, and methods such as excluding individual articles one by one and changing analysis methods were adopted to re-examine the size and changes of the effect size. The results show that excluding individual studies does not have a significant impact on the outcome measures, indicating that the meta-analysis results of this study have a certain level of credibility.

## DISCUSSION

### Pathogenesis of ADHD

#### Physiological mechanism of neurotransmitter abnormalities mediating ADHD lesions

The occurrence of ADHD is mainly related to abnormal monoamine neurotransmitters. Monoamine neurotransmitters are involved in the occurrence of ADHD and its related diseases, such as conduct disorders and oppositional resistance disorders. The pathological characteristics of ADHD are controlled by neurotransmitters such as dopamine (DA), norepinephrine (NE), and serotonin. Abnormal monoamine neurotransmitters directly mediate the core symptoms of ADHD patients [33,34]. Research has shown that the occurrence of ADHD is closely related to dopamine imbalance, and its pathway plays an important role in attention and memory, mainly influenced by neural impulses affecting reward mechanisms and responses to external stimuli [35]. This is closely related to aggression, impulsivity, depression, emotion regulation, brain area activity and cell survival, brain development, and various neurological and psychiatric disorders [34,36].In addition, the dopaminergic system plays a crucial role in maintaining the pathogenesis of ADHD, relying on its bottom-up-regulation of reward processing. Adult ADHD may experience functional impairments in processing sensory inputs and recognising which inputs are worth noting when fixed [6]. Affects an individual's motor control, and cognitive ability regulation [37]. However, the decrease in alpha 2A receptors for norepinephrine is associated with a decrease in norepinephrine concentration in the synaptic cleft of neurons [38,39]. The dysfunction of the noradrenergic system is an important cause of ADHD brain dysfunction. Meanwhile, catecholamines are closely related to dopamine. Dopamine is the most active neurotransmitter of catecholamines, and its secretion can affect the transmission of damaged neurotransmitters, thereby altering individual behavioural activities and physiological rhythms. Damage to the prefrontal cortex of ADHD leads to reduced secretion of dopamine receptors, which in turn causes catecholamine dysfunction [40].In contrast, the 5-HT system in the brain largely mediates impulses and emotional abnormalities, and changes in its neural transmission can lead to impulsive, aggressive, and hyperactive behaviour. Vanicek et al. found significant differences in the expression of 5-HT transporters in the anterior cingulate cortex and hippocampus of ADHD patients compared to the normal population, indicating that the coupling ability of 5-HT transporters in the anterior cingulate cortex and hippocampus may have been altered [41]. In addition, the decrease in 5-HT levels in ADHD patients increases their aggressive behaviour, and there is an abnormal correlation between the right anterior motor area the left somatosensory area, and the default mode network functional connectivity [42]. In summary, dopamine system disorders decreased norepinephrine concentration, and changes in 5-HT activity involved in the abnormality of monoamine neurotransmitters are important physiological mechanisms mediating the onset of ADHD.

#### Biological mechanisms of ADHD lesions mediated by abnormal brain structure and activation patterns

The pathogenesis of ADHD may be related to individual changes in brain volume, abnormal subcutaneous structures, and impaired function of the prefrontal cortex [43]. Meanwhile, in adults diagnosed with ADHD, brain function and structural disorders involve executive function, reward expectations, and attention processes. The neurobiological theory of ADHD suggests that late-maturing brain regions, executive dysfunction, and metabolic disorders may be the basis for some of the neurocognitive deficits observed in ADHD, such as attention and impulse control. The theory of executive dysfunction suggests that deficits in multiple domains associated with ADHD are caused by cognitive dissonance in the ‘higher-order’ central executive system [44].

Based on functional magnetic resonance imaging (fMRI) research, it has been found that the ventral lateral prefrontal cortex and insula striatum regions of ADHD patients are small and dysfunctional, revealing structural and functional defects in the prefrontal cortex and striatum of ADHD patients. Meanwhile, dopamine deficiency in the prefrontal and subcortical regions involves three main pathways: the midbrain limbic pathway, the midbrain cortex pathway, and the nigrostriatal pathway [36]. Therefore, ADHD is closely related to the prefrontal cortex. Meta-analysis found that ADHD patients have reduced gray matter volume in the frontal-parietal region, limbic system, and corpus callosum region. In addition, significant low activation was found in several frontal and temporal brain regions, right central posterior gyrus, left insula, and corpus callosum, suggesting that abnormal changes in the structure and function of the left frontal gyrus and corpus callosum may be key to the pathogenesis of ADHD in patients [45]. Grey matter volume in the precentral gyrus, medial orbitofrontal cortex and (accessory) cingulate cortex was significantly smaller in ADHD patients [46]. From the changes in white matter volume in individuals with ADHD, it can be observed that the white matter regions of the primary motor cortex, as well as the anisotropic scores in the frontal striatal thalamus and frontal-parietal lobe circuits, are reduced in adult ADHD [47]. A recent longitudinal diffusion imaging study of individuals from childhood to adulthood found that white matter microstructures develop faster, including the arcuate tract, cingulate gyrus, a superior longitudinal tract of the interfrontal occipital tract, oblique frontal tract, and frontal striated tract connecting the prefrontal cortex [48]. Adult ADHD patients who have not received medication treatment exhibit increased local connectivity in the dorsal anterior cingulate cortex and superior frontal gyrus, followed by decreased local connectivity in the posterior cingulate cortex [49]. Therefore, there are differences in gray matter volume, white matter microstructure, and cortical thinning in ADHD patients, while the primary motor cortex, frontal lobe, and neck also have certain functional correlations. In addition to structural abnormalities in the brain, functional disorders of the cerebral cortex in patients with ADHD are also one of the important mechanisms underlying the onset of the disease. Functional magnetic resonance imaging analysis shows that multiple frontal and temporal brain regions in individuals with ADHD are in a significantly low activation state, including the right central posterior gyrus, left insula, and corpus callosum [45]. The insufficient activation of these brain regions may be related to the negative impulses and inhibitory behaviours observed in adult ADHD symptoms. However, overactivated brain regions include the occipital gyrus, right insula, right anterior cingulate cortex, right caudate nucleus, right central operculum, and sensory-motor network [50]. Overactivation of these brain regions can lead to dysfunction of executive function and attention control networks [51].

### Possible mechanisms by which physical activity improves inhibitory control of adult ADHD

#### Physical activity regulates the structure and function of the prefrontal cortex

Physical activity can enhance brain plasticity, thereby improving the structure and function of the prefrontal cortex. The prefrontal cortex (PFC) serves as the neural basis for inhibitory control and is primarily responsible for interfering with advanced cognitive functions such as inhibition, conflict resolution, and selective attention. The neurons in the prefrontal cortex are mainly distributed in the right dorsolateral prefrontal cortex and frontal polar region. At the same time, the function of the prefrontal cortex varies in different regions, and excitatory neurons are mainly scattered in the anterior cingulate gyrus. The anterior cingulate gyrus has a regulatory effect on inhibitory control neurons [52]. When the current cingulate gyrus, lateral prefrontal cortex, and frontal pole are activated and enhanced together, the inhibitory control function will be enhanced as the demand for supportive attention control increases [53]. And studies have shown that the activity of the right dorsolateral prefrontal cortex in ADHD patients who participate in long-term exercise is significantly higher than that in ADHD patients who do not exercise [54]. Therefore, the enhancement of inhibitory control depends on the dynamic interaction between the prefrontal cortex and other cortical regions. From the perspective of the positive adaptability of physical activity to the prefrontal cortex, research has shown that physical activity can increase the volume of the prefrontal cortex, promote more effective task-related activation of the prefrontal and motor networks, and provide more replenishment of prefrontal brain resources [40].In addition, acute exercise may have a priming effect on the central nervous system by briefly increasing blood flow in the prefrontal cortex, basal ganglia pathway, cingulate gyrus, and caudate nucleus regions [55]. The total amount of hippocampus, gray matter, and white matter in the brain increases, improving information transmission and cerebral blood flow between different regions of the brain. Oxygen supply to the frontal lobe of the brain increases, and the regenerative ability of nerve cells is enhanced, thereby promoting the development of inhibitory control [56]. Therefore, physical activity improves inhibitory control ability by increasing the capacity of the prefrontal cortex and the number of its receptors, thereby enhancing the prefrontal cortex function of ADHD.

#### Physical activity regulates neural activation levels in specific brain regions

With the advancement of science and technology, the level of brain activation can be observed through techniques such as brain imaging. The improvement of brain activation patterns is an important manifestation of brain remodeling, indicating that brain function and cognitive function will also change. Through fMRI, not only can the processing of specific brain regions during cognitive tasks be observed, but also the signal time series correlation of blood oxygen dependence levels in multiple brain regions can be detected, that is the brain functional connectivity pattern [57]. Long term participation in physical activity can affect the activation level of advanced cognitive function areas in the brain. Research has shown that the prefrontal cortex (*i.e.* the orbitofrontal and dorsolateral regions) increases activation levels during slow and fast movements, which in turn affects inhibitory control and working memory [58]. Up-regulation of activation levels in the striatum and insula is beneficial for motor-related learning abilities and attention control [59]. Acute aerobic exercise enhances the activation levels of the individual cerebral cortex (frontal lobe, temporal lobe, parietal lobe, and occipital lobe) and subcortical regions (thalamus, amygdala, hippocampus, and cerebellum), revealing that the improvement benefits of exercise on working memory are related to changes in brain activation patterns [60]. In addition, when individuals complete complex tasks, they not only need changes in the activation levels of specific brain regions but also require coordination and connectivity of multiple brain region functions. Exercise can improve the functional connectivity of specific brain regions in advanced cognitive function. For example, physical activity improves the functional connectivity between the right cerebellum and the right inferior frontal gyrus, thereby enhancing their inhibitory control [61]. The degree of functional connectivity between the thalamus and striatum can predict an individual's level of executive function [59]. In addition, exercise induces activation of single and multiple brain regions to improve inhibitory control ability. For example, the activation patterns of functional networks such as the frontal lobe executive control network, default network, auditory-related network, *etc*. The changes in the activation of these networks may be the core mechanism for improving inhibitory control [62]. It can be seen that under the intervention of exercise, changes in brain activation patterns are accompanied, thereby optimising inhibitory control.

### Positive benefit analysis of physical activity improving inhibitory control of adult ADHD

This study conducted a meta-analysis on the impact of physical activity on inhibitory control in adult ADHD patients. The results indicate that physical activity has a positive effect on the inhibitory control of adult ADHD. At the same time, there is a certain relationship between the improvement of inhibitory control ability and the type of physical activity and the exercise programmes involved in physical activity, which is consistent with previous research conclusions [63]. Numerous studies have found that physical activity is one of the effective means for cognitive function in children and adolescents with ADHD [40,64]. However, there is relatively small amount of literature on the effects of physical exercise on adults with ADHD. In existing research, participation in leisure sports activities can improve cognitive and behavioural performance related to attention, inhibition, motivation, and impulsivity [65]. The view that physical activity improves individual physical function under the concept of ‘exercise is good medicine’ has become a consensus. Ogrodnik et al. (2023) found that higher cardiorespiratory fitness levels in adult ADHD are positively correlated with inhibitory control levels [66]. Physical activity is a potentially effective intervention measure with high compliance, tolerability, and feasibility for intervening in the core symptoms of adult ADHD [67]. A review study found that both open and closed exercises improved executive function in ADHD patients and encouraged them to participate in physical activities involving multiple types of motor skills [68]. In addition, resting-state fMRI reports that aerobic exercise mediates spontaneous activation and changes in the left, middle, and right frontal gyrus of ADHD, which are directly related to the development of attention and inhibitory control in these two prefrontal cortex regions [69]. In summary, physical activity is an effective intervention for improving ADHD inhibitory control, which is consistent with current and previous research findings.

From the analysis of different subgroups of exercise types, the improvement effect of chronic exercise on the inhibitory control ability of ADHD is better than that of acute exercise. However, this conclusion is limited by the quantity of research evidence and still needs to be substantiated. The reasons for this inconsistency may be due to different exercise prescriptions, and dose effects, as well as the different pathological types, physiological characteristics, and daily medication interventions of ADHD patients. But when research suggests that different types of exercise have a positive effect on the inhibitory control ability of ADHD. A retrospective study found that there are certain differences in the benefits of aerobic exercise on individual cognitive function across different cycles. Acute aerobic exercise can effectively improve individual inhibitory control and attention, while short-term and medium to long-term aerobic exercise have a positive effect on neural networks and memory function. However, overall, adhering to regular aerobic exercise can effectively improve individual cognitive function [70]. The neurophysiological results during acute exercise showed that under moderate and high-intensity aerobic exercise intervention conditions, the short-interval cortical inhibition in the adult ADH brain significantly increased, revealing a positive correlation between changes in short-interval cortical inhibition and improvement in inhibitory control [71]. After the intervention of acute aerobic exercise in ADHD college students with executive function deficits, it was found that although the improvement benefits of sub-components such as working memory and cognitive flexibility in executive function were not significant, they positively improved the patient's inhibitory control ability level [72].

However, some studies suggest that physical activity does not directly improve the cognitive function level of adult ADHD. The benefits of improving core symptoms such as attention span and impulsivity may be achieved through different pathways and methods to enhance their cognitive performance [73]. From the perspective of chronic exercise, randomised controlled trials are relatively rare, which may be limited by the specific characteristics of adult patients, work and life pressures, and uncontrollable experimental design factors. Research has shown that an increase in weekly energy expenditure during adolescence is significantly correlated with a decrease in ADHD symptom levels in early adulthood, revealing that physical activity may be a protective factor for the onset of ADHD in patients [74]. Moderate or high-intensity exercise with an intervention period lasting more than 12 weeks and an intervention frequency exceeding twice a week may be an appropriate exercise dose for individuals with ADHD [68]. In addition, studies have shown that moderate-intensity physical activity lasting for more than five weeks, with a frequency of three times a week and 30 minutes each time, will further improve the levels of attention, inhibition, emotional control, behaviour, and exercise control in ADHD [75]. Regular participation in physical activity cannot only effectively alleviate the complications of ADHD and obesity in patients, but also improve brain function, regional and biomarker deficiencies, as well as hypothalamic pituitary adrenal dysfunction, thereby improving the health behaviour and quality of life of ADHD [76]. A 12-week (three times a week) mixed exercise programme that includes aerobic exercise, strength, and flexibility exercises. After an intervention, it was found that the core ADHD symptoms, quality of life, physical awareness, sleep, and cognitive function of patients all had positive effects, and ADHD patients had high compliance, revealing the feasibility and effectiveness of regular physical activity participation in adult ADHD [77]. In summary, both acute and chronic exercise have a positive effect on the inhibitory control ability of ADHD, which is consistent with previous research findings.

From the perspective of different exercise programmes, it was found that Pilates produced the maximum effect size and had the best improvement effect on the inhibitory control ability of adult ADHD. However, only one piece of literature was included for analysis, and the conclusions drawn from the summary analysis of a few pieces of literature need to be examined. Next is the cycling group, which has included a relatively large number of studies and conclusions with higher credibility. It can be concluded that the cycling group is superior to other forms of exercise. In addition, the yoga group had a lower effect size but still had an improved effect on the inhibitory function of adult ADHD. Finally, the vibration training and Tai Chi group have a certain level of effect, but not significant. Only one article was included for analysis, and the conclusions drawn from the inductive analysis of a few articles may be biased, which needs further investigation in the future. Analysis has found that participating in power cycling exercises not only improves physiological arousal levels but also does not produce internal noise. Therefore, power cycling is often used as a common intervention for acute aerobic exercise [78]. Research has revealed that both sustained moderate-intensity aerobic exercise and high-intensity interval training positively impact individual cognitive function, particularly in terms of enhancing conflict suppression. However, their behaviours and neurophysiological manifestations differ. The former elevates an individual's brain arousal level, whereas the latter potentially enhances conflict suppression performance by accelerating cognitive refresh rates [79]. In addition, the relatively novel concept of exergaming serves as a personalised home intervention programme for ADHD patients, effectively enhancing their inhibitory control abilities [80]. Among various intervention methods, ball sports have been identified as an effective way to enhance inhibitory control in ADHD patients, followed by physical and mental exercises, cognitive task-based exercises, and single aerobic exercises [64]. Different physical activity modes exert varying impacts on ADHD patients. Specifically, cognitive-motor training demonstrates the most significant improvement in inhibitory control, aquatic exercises yield the greatest enhancement in attention and cognitive flexibility, while aerobic activities exhibit the most beneficial effects on working memory [81]. The conclusions of the current study are not entirely consistent with previous research, possibly due to limitations in the research literature. Additionally, the impact of different physical activity interventions on inhibitory control in adult ADHD may vary depending on the individual and the effectiveness of the intervention. Therefore, it is impossible to determine which sports offer the best improvement in inhibitory control ability for adult ADHD.

### Limitations

This article still has certain limitations. First, most of the studies included in this paper did not mention allocation concealment and blinding, and there is some risk of bias; researchers or subjects may participate selectively or pay more attention to subgroups because they know about them, and raters may be biased, all of which can lead to biased results and affect the reliability of the conclusions. Second, since most of the study outcome indicators were dominated by inhibitory control, future studies could expand the range of outcome indicators to include working memory and cognitive flexibility. Third, considering the influence of cultural or geographic factors on physical activity patterns and ADHD symptoms may help explain differences in findings across populations. Fourthly, owing to the limited availability of research materials, this paper solely incorporates English-language literature, which may potentially compromise the comprehensiveness of the data to some extent. In the subgroup analysis, there is a limited amount of literature that incorporates chronic exercise and various physical activity programmes. It is anticipated that further research will be conducted in the future to enrich the meta-analytic results in this area, providing effective evidence for exploring the positive benefits of physical activity on the cognitive function of adults with ADHD.

## CONCLUSIONS

Physical activity exerts a positive effect on inhibitory control in adults with ADHD. Different types of exercise exhibit beneficial effects on ADHD inhibitory control, among which chronic exercise demonstrates superior improvements compared to acute exercise. However, this conclusion remains to be validated due to the limited number of studies. In subgroup analyses, the research literature on chronic exercise and specific physical activity programmes is sparse. Future studies should control for change in adult ADHD status or baseline ADHD severity to more scientifically explore the effects of physical activity on inhibitory control. Although rational, optimised exercise intervention protocols continue to be refined, this does not preclude the recommendation of moderate- or high-intensity, acute, long-term, multiple exercise modalities as potential therapeutic tools for the treatment of inhibitory control in adults with ADHD.

## Additional material


Online Supplementary Document
