# Supplementary material for: The impact of physical activity on inhibitory control of adult ADHD: a systematic review and meta-analysis
Source: J Glob Health. 2025 Mar 14;15:04025. doi: 10.7189/jogh.15.04025 (PMC11907377; doi:10.7189/jogh.15.04025)

Supplement to: Yang Y, Wu CH, Sun L, Zhang TR, Luo J. The impact of physical activity on inhibitory control of adult ADHD: a systematic review and meta-analysis. J Globh Health. 2025;15:04025.

Figure S1. Meta analysis forest plot of the effects of different types of exercise on inhibitory control of adult ADHD.

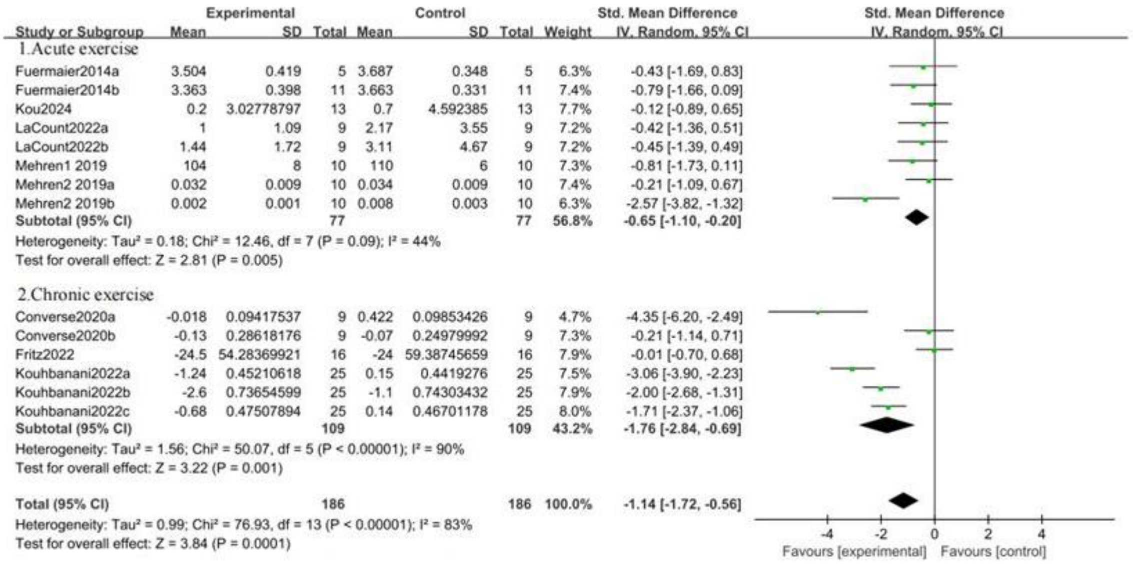

Figure S2. Meta analysis forest plot of the effects of different exercise programs on inhibitory control of adult ADHD.

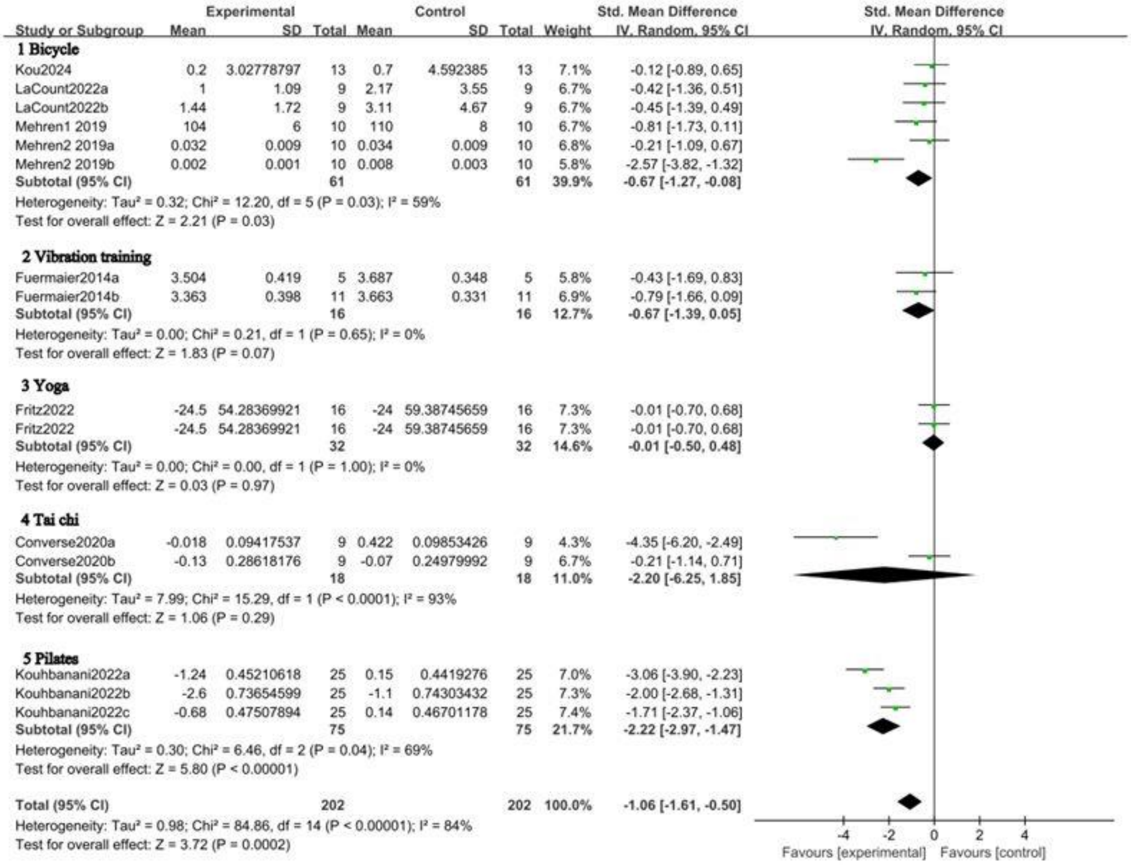

Figure S3. Publication bias in literature on the impact of physical activity on inhibitory control of adult ADHD.

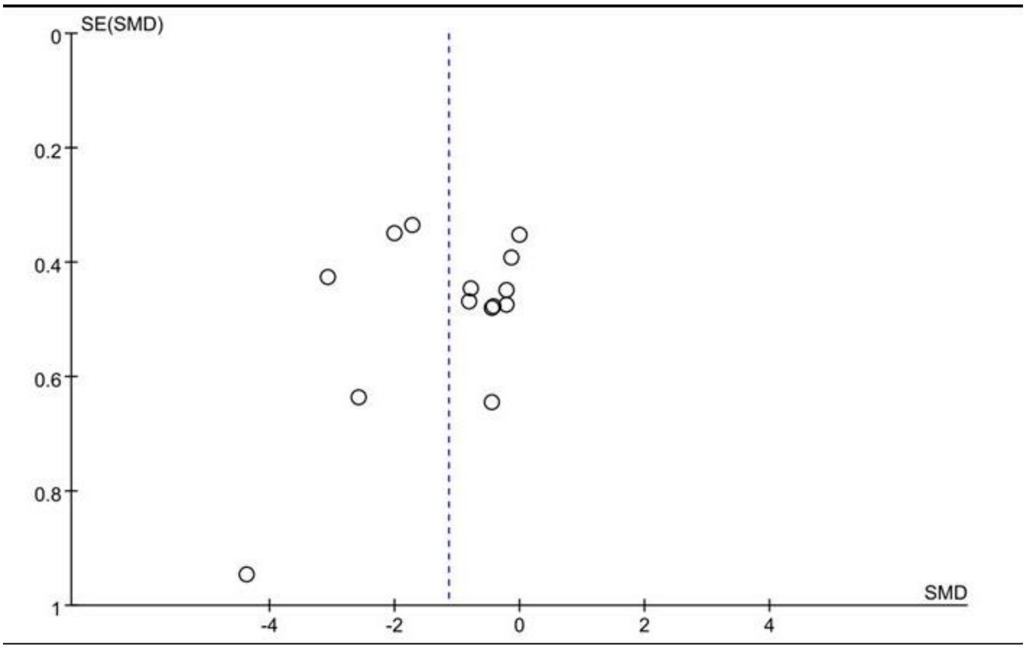

Supplement: Online Supplementary Document [file jogh-15-04025-s001.pdf]
